# Data-Efficient Transformer Model for Retinal Artery-Vein Segmentation in Landmark-Deficient OCT Angiography

**DOI:** 10.1016/j.xops.2026.101416

**Published:** 2026-09-23

**Authors:** Cyriac Manjaly, Haneen Alfauri, Aditya Santoki, Nicholas Kikuta, Lauren Pollack, Senyue Hao, Chao Zhou, Rithwick Rajagopal

**Affiliations:** 1 Department of Ophthalmology and Visual Sciences, Washington University School of Medicine, St. Louis, Missouri; 2 Department of Electrical & Systems Engineering, Washington University in St. Louis, St. Louis, Missouri; 3 John A. Burns School of Medicine, University of Hawaiʻi at Mānoa, Honolulu, Hawaii; 4 Saint Louis University School of Medicine, St. Louis, Missouri; 5 Department of Biomedical Engineering, Washington University in St. Louis, St. Louis, Missouri

**Keywords:** OCT angiography, Artery-vein segmentation, Deep learning, Retinal vein occlusion, Data-efficient learning

## Abstract

**Objective:**

Accurately differentiating arteries from veins in highly magnified foveal 3 × 3-mm^2^ macular OCT angiography (OCTA) is critical for quantifying retinal vascular disease. However, existing models require macrovascular landmarks absent at this field of view, hindering vessel-specific analysis in clinical imaging. We developed and validated a data-efficient, 2-stage transformer for artery-vein (AV) segmentation in landmark-deficient macular OCTA and evaluated whether the resulting vessel maps could identify retinal vein occlusion (RVO) biomarkers without disease-specific retraining.

**Design:**

Cross-sectional model development and validation study.

**Participants:**

The development cohort comprised 17 eyes (29 scans) from 9 healthy adult volunteers (mean age, 32 years). A subsequent RVO pilot cohort included 9 foveal OCTA scans from patients with confirmed RVO, compared against 15 healthy control scans.

**Methods:**

Independent 2-stage Swin-Unet transformer trained on sparse annotations from 12 eyes, applied without retraining to 5 test eyes and the RVO pilot cohort.

**Main Outcome Measures:**

Artery-vein segmentation Dice coefficients, area under the receiver operating characteristic curve (AUC), sensitivity, and specificity. Clinical RVO end points included the major vein-to-artery sinuosity ratio and the 10th-percentile artery radius.

**Results:**

The framework demonstrated exceptional internal validation and crucially, when applied to the external RVO cohort, the model successfully identified established pathophysiological hallmarks. Stage 1 global segmentation achieved AUC 0.973 and Dice 88.6%. Stage 2 AV classification achieved Dice 96.3% (arteries) and 96.1% (veins). End-to-end system Dice coefficients were 87.3% and 84.1% for arteries and veins, respectively. Applied to RVO without retraining, the model identified significantly elevated major vein/artery sinuosity (1.041 vs. 1.010; *P* = 0.007; AUC, 0.837) and reduced 10th-percentile artery radius (13.2 vs. 15.4 μm; *P* = 0.002; AUC, 0.885).

**Conclusions:**

This data-efficient framework enables high-fidelity AV segmentation in landmark-deficient OCTA. By combining conventional methods with newer transformer models and decoupling structural segmentation from classification, the model identified biologically consistent biomarkers in RVO from small sets of scans. This supports highly scalable automated vessel specific quantification in clinical macular imaging; these pilot findings are hypothesis-generating and warrant prospective validation.

**Financial Disclosure(s):**

Proprietary or commercial disclosure may be found in the Footnotes and Disclosures at the end of this article.

The retina serves as a unique window into systemic health,^1^, ^2^, ^3^, ^4^, ^5^, ^6^ allowing for noninvasive visualization of the microvasculature. OCT angiography (OCTA) has revolutionized this field^7^^,^^8^ by providing high-resolution and depth-resolved images of the retinal capillary network. Differentiating retinal arteries from veins is important in the diagnosis and monitoring of diseases such as diabetic retinopathy^6^^,^^9^, ^10^, ^11^ and retinal vein occlusion (RVO),^12^, ^13^, ^14^ where vessel-type-specific changes correlate directly with visual prognosis.^11^^,^^15^ In RVO, for example, venous outflow obstruction drives selective venous tortuosity, whereas peripheral arteriolar narrowing reflects the coexisting hypertensive and arteriosclerotic disease commonly associated with RVO. These vessel-type-specific changes are invisible to metrics that aggregate both systems.^12^^,^^13^

In standard widefield imaging, differentiation is often guided by clear morphological hierarchy.^16^, ^17^, ^18^ However, foveal-centered 3 × 3-mm^2^ macular scans lack these global landmarks.^19^^,^^20^ At this high magnification, the field of view captures only fourth- and fifth-order vessels alongside a dense capillary mesh, making traditional feature-based differentiation ineffective.^18^^,^^21^, ^22^, ^23^ Deep learning offers a promising solution, yet existing single-stage approaches at this resolution attempt to simultaneously segment and classify vascular structures in a unified step and are trained on data that includes reliable landmarks or multiple imaging modalities.^14^^,^^19^^,^^23^, ^24^, ^25^, ^26^ These end-to-end architectures typically require hundreds to thousands of annotated images to achieve clinical fidelity, relying heavily on first- and second-order vascular landmarks (such as the major arcades) for reliable spatial orientation cues. In foveal-centered 3 × 3-mm^2^ macular OCTA scans, however, only fourth- and fifth-order vessels are captured within a dense capillary mesh lacking a predictable anatomical pattern. Without these macrostructural guides, the distinct caliber and orientation differences of larger vessels vanish, causing traditional models to struggle with vessel continuity and accurate artery-vein (AV) differentiation. This challenge is further compounded by a scarcity of high-quality expert annotations, as the labor-intensive nature of manually tracing complex capillary networks remains a significant bottleneck for developing robust automated tools.^24^^,^^27^

Recent work has specifically addressed AV segmentation in 3 × 3-mm OCTA. Abtahi et al^19^ introduced MF-AV-Net, a convolutional neural network (CNN) with multimodal fusion options trained on 150 annotated images, and Sadhin et al^28^ subsequently developed OCTA-ReVA + AV, an open-source toolbox built upon MF-AV-Net. While these represent valuable contributions, several considerations motivate additional methodological development. First, both systems rely on CNN architectures that build global image context indirectly through downsampling with skip connections. Transformer architectures directly capture global context through self-attention; an advantage we hypothesize becomes particularly important in pathological states such as RVO, where disease-specific vascular changes are best recognized in their global context. Second, both employ single-stage pipelines that must simultaneously segment and classify vessels, whereas decoupling these tasks allows specialized optimization of each objective. Third, we explicitly avoid assigning capillary-level vessel identity due to the lack of a biological consensus. Prior work generally tends to assign capillary identity based on whether the nearest vessel is an artery or vein. However, for pathological exploration where morphological vessel measures such as tortuosity vary, nearest neighbor assignments may bias downstream measurements.

To this end, and to address prior limitations, we introduce a data-efficient, 2-stage deep learning framework for automated AV differentiation in landmark-deficient macular OCTA. By training a Swin-Unet^29^ with a lenient Frangi filter prior, we combine the long-range context modeling of transformers^25^^,^^26^^,^^29^ with the precision of traditional computer vision.^30^^,^^31^ This 2-stage approach explicitly decouples structural segmentation from vessel classification, achieving high-fidelity performance using a training set of only 12 eyes. As a proof of concept, we further evaluate whether the resulting vessel maps can recover prespecified vessel-type-specific biomarkers in a pilot RVO cohort without disease-specific retraining.

## Methods

This study was approved by the Washington University School of Medicine Institutional Review Board and adhered to the Declaration of Helsinki. Informed consent was obtained from all participants.

### Study Population and Data Acquisition

This cross-sectional study recruited 9 healthy adult volunteers (6 males, 3 females; mean age, 32 years) from the Department of Ophthalmology at Washington University in St. Louis. The development cohort consisted of 17 eyes (29 total scans). All 17 eyes were imaged in the dark-adapted state, yielding 1 baseline scan per eye. A randomly selected subset of 12 eyes was additionally imaged immediately following a brief light stimulus, yielding 1 poststimulus scan per eye. The purpose of including these scans was to introduce natural temporal variation in the training data, improving model robustness and scan-to-scan variability; the expected physiological changes of transient capillary flow alterations are not captured at the spatial resolution of OCTA and do not alter the morphological features used for AV classification. Partitioning was performed strictly at the eye level to prevent data leakage; all scans belonging to a single eye were assigned to the same partition. Eyes were randomly assigned to training or holdout test sets at a 2-to-1 ratio. This single train-test split is a deliberate design choice consistent with the central thesis of this work: demonstrating that clinically useful AV segmentation for pathological exploration can be achieved by training a model once on a small set of annotated data and applying it without modification to unseen eyes and disease states. A holdout test set was utilized: with only 9 participants, participant-level k-fold cross-validation would produce folds containing as few as one to 2 participants in the test set, yielding highly unstable per-fold estimates that may not capture true model variability. Instead, uncertainty was quantified using standard bootstrap resampling (N = 2000 iterations) of test-set predictions to generate 95% confidence intervals (CIs).

### Ground Truth Generation

To link the macular microvasculature to the optic disc, we utilized near-infrared–mediated mapping.^32^ Because small-field OCTA lacks global landmarks, we used coregistered near-infrared confocal scanning laser ophthalmoscopy as an intermediate reference to widefield color fundus photography. Two independent graders manually traced the vascular network on color fundus photography, starting at the optic disc where major arcades are readily distinguishable, and propagated identities distally. Discrepancies were resolved by a retinal physician (R.R.). These labels were mapped back to OCTA via the near-infrared reference. Annotations were intentionally sparse; dense capillary meshes remained unannotated to avoid introducing uncertain training data, necessitating masked loss functions during training. At vessel crossings, where an artery passes over or under a vein in the en face OCTA projection, both vessels were labeled at the crossing point, creating a region represented by the “overlap” class.

### Two-Stage Deep Learning Pipeline

We developed a 2-stage deep learning pipeline in PyTorch using a Swin-Unet transformer backbone to explicitly decouple structural vessel segmentation from physiological classification. As shown in Figure 1, Stage 1 segmented the vascular network from background capillary noise. Although various techniques exist for enhancing tubular structures, segmentation approaches such as Frangi-based segmentation alone degrade at the 3 × 3 mm due to excessive false positives from capillaries and necessitate a deep learning approach. To preserve the global contextual connections necessary to trace disjointed vessels across the macula, images were processed in a single forward pass over the full field of view rather than using patch-based sliding windows. The input comprised a 2-channel tensor: the raw grayscale OCTA signal and a geometric prior. This prior utilized contrast-limited adaptive histogram equalization and a specialized Frangi vesselness filter, which was found to enhance performance compared to approaches not utilizing a geometric prior. To ensure reproducibility and prevent data leakage, the filter parameters were not optimized on the training or test datasets; instead, they were fixed to be as lenient as possible to maximize sensitivity for both thick vascular trunks and faint capillary segments based on established literature values.

**Figure 1:**
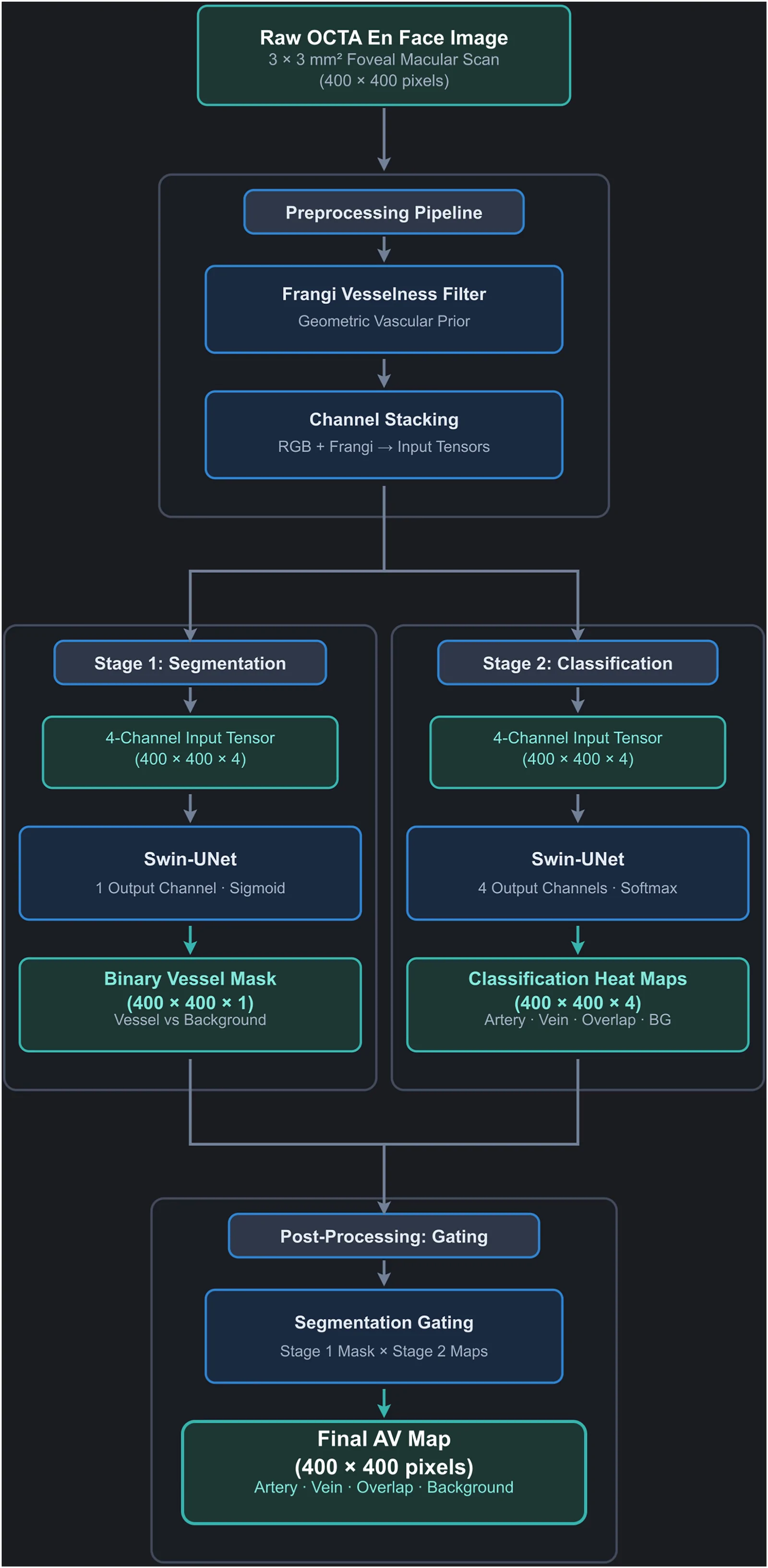
Algorithm development: 2-stage framework. General schematic of the 2-stage data-efficient transformer framework for automated AV segmentation. The raw OCT angiography en face image (3 × 3 mm^2^) first passes through a preprocessing pipeline, in which Frangi vesselness filtering and channel stacking (RGB + Frangi) enhance vascular features. In Stage 1 (segmentation), a Swin-Unet generates a single-channel binary vessel mask (vessel vs. background) using sigmoid activation. In Stage 2 (classification), a second Swin-Unet uses a 4-channel input to produce classification heat maps for 4 categories (artery, vein, overlap, and background) via softmax activation. In the final postprocessing step, the Stage 1 binary mask gates the Stage 2 heat maps to assign physiological identities only to segmented vascular regions. AV = artery-vein; BG = blue green; RGB = red, green, and blue.

Stage 2 assigned physiological identities (artery, vein, or overlap) to the pixels isolated in Stage 1. This classifier utilized a 4-channel input strategy, combining the Frangi map with the OCTA intensity replicated across 3 color channels to leverage pretrained ImageNet weights. To handle the sparse annotations, a masked loss function combining weighted cross-entropy and Dice loss restricted gradient updates exclusively to high-confidence expert labels. Dynamic class weighting was applied to address anatomical class imbalances. Both stages were optimized using the Adam optimizer with cosine annealing and an early-stopping mechanism to prevent overfitting. The complete pipeline required approximately 180 min to train and achieved an average inference time of 0.10 s per scan.

To optimize model performance and training stability, both stages were trained for a maximum of 1000 epochs. An early-stopping mechanism was employed to monitor validation loss, with a patience threshold of 100 epochs. If validation loss failed to improve within this window, training was terminated to prevent overfitting to the limited training cohort. Stage 1 (Segmentation) reached convergence at Epoch 754, while Stage 2 (Classification) converged at Epoch 385. This difference in convergence speed is attributed to the distinct complexity of the tasks: Stage 1 requires learning global vascular connectivity from noise-heavy background, whereas Stage 2, having already received a binarized vessel map, focuses on local physiological classification.

Hyperparameter tuning was performed via grid search on the training set. For both stages, we utilized a learning rate of 3 × 10^–5^ combined with a cosine annealing scheduler (T_max_ = 5000), which facilitated a gradual decay in step size as the model approached convergence. This scheduler was particularly effective in allowing the model to escape suboptimal local minima in the early phases of training before settling into a robust feature representation. The masked loss function was pivotal for handling our sparse annotations; by nullifying the contribution of pixels without ground-truth labels, the model was forced to learn exclusively from high-confidence vascular features. To further regularize the model, we implemented a weight decay of 1 × 10^–4^ and dropout layers (rate = 0.2) within the transformer blocks. These strategies collectively ensured that the model remained robust to the inherent variations in small-field OCTA imaging, such as localized signal attenuation and variation in capillary density, which often confound traditional architectures.

### Proof-of-Concept Application: RVO

To evaluate whether the trained pipeline extracts clinically meaningful biomarkers without additional fine-tuning, we applied the fixed model to a pilot cohort of 9 patients (9 high-quality scans) with confirmed RVO. All included RVO patients had a concurrent history of hypertension. This cohort was compared against a healthy control group of 15 high-quality images. Other control images acquired at different fields of view were excluded to avoid resolution confounds. To assess segmentation and classification accuracy on pathological data, expert AV ground truth was generated in the same manner as the holdout test set for all 9 RVO scans. This biomarker analysis is exploratory and hypothesis-generating; it does not constitute a formal diagnostic accuracy study.

### Vascular Biomarker Extraction

With the model weights strictly held constant, morphological biomarkers were extracted from the predicted AV maps via skeletonization and connected-component branch analysis. The binary vessel mask for each vessel type (artery, vein) was skeletonized to a 1-pixel-wide centerline, and the skeleton was parsed into individual branches using junction detection: skeleton pixels with more than 2 neighbors were identified as junction points, and branches were defined as connected skeleton segments between 2 junction points or between a junction point and a terminal end point. Branches shorter than 20 pixels (approximately 62 μm or 5% the length of the image) were discarded as skeleton pruning artifacts rather than true vessel segments.

Sinuosity was defined as the ratio of actual vessel path length (measured along the skeleton pixel-by-pixel) to the Euclidean straight-line distance between the branch's 2 end points (sinuosity = path length/chord length). A perfectly straight vessel has a sinuosity of 1.0. To prevent short branches from dominating the estimate, sinuosity was computed globally as the sum of all branch path lengths divided by the sum of all branch chord lengths, rather than as a per-branch average.

Vessel radius was computed via the Euclidean distance transform applied to the binary vessel mask. For each skeleton pixel, the distance transform value equals the distance to the nearest background pixel, equivalent to the radius of the largest inscribed circle at that point. Branch-level radius was summarized as the median radius along each branch skeleton. Population-level summaries included the mean radius, median radius, and the 10th-percentile radius (p10), which captures the typical width of the thinnest vessel segments.

Two prespecified morphological end points were extracted: the major AV sinuosity ratio (restricted to branches ≥310 μm to isolate larger vessels) and the 10th-percentile artery radius. These end points were selected to directly reflect established RVO pathophysiology. The sinuosity ratio captures the selective venous tortuosity caused by outflow obstruction while utilizing the patient's own arteries as self-standardizing internal control. Conversely, the 10th-percentile artery radius isolates the specific narrowing of the smallest peripheral arterioles, a subtle morphological change that is typically obscured when evaluating mean vessel caliber. Group comparisons were conducted using 2-sided Mann–Whitney *U* tests, with the area under the receiver operating characteristic curve (AUC) evaluated via bootstrap resampling over 2000 iterations.

## Results

The proposed 2-stage framework was evaluated on a test set comprising 10 OCTA scans from 5 eyes. Performance metrics are summarized in Table 1. All 95% CIs in Table 1 were derived from bootstrap resampling (*N* = 2000 iterations) of test-set predictions. Performance was assessed at 4 levels: Stage 1 vessel segmentation, Stage 2 AV classification within ground truth vessel regions, the combined end-to-end system, and its performance against RVO vein/artery tortuosity and arterial thinning end points. The segmentation model achieved robust detection of the retinal vascular network, distinguishing fourth- and fifth-order arterioles and venules from the background capillary mesh. The model achieved a Dice coefficient of 88.6% with an AUC of 0.973, demonstrating high discriminative capacity for vessel detection despite the absence of macrovascular landmarks. Specificity was notably high at 0.986, indicating minimal false-positive vessel detection in the background.

**Table 1:** Performance Metrics for Vessel Segmentation and Artery-Vein Classification

Task/Class	Dice Score (95% CI)	AUC (95% CI)	Accuracy (95% CI)	Sensitivity (95% CI)	Specificity (95% CI)
Stage 1: vessel segmentation	88.6% [87.1–90.1]	0.973 [0.963–0.983]	96.7% [96.2–97.3]	86.4% [83.0–89.8]	98.6% [98.3–98.9]
Stage 2: artery classification	96.3% [95.1–97.5]	0.991 [0.986–0.996]	96.3% [95.1–97.5]	95.0% [92.5–97.5]	97.8% [96.7–98.9]
Stage 2: vein classification	96.1% [94.8–97.4]	0.992 [0.988–0.996]	96.0% [94.8–97.3]	98.0% [97.0–99.1]	94.3% [91.7–96.9]
End-to-end: artery segmentation and classification	87.3% [85.7–88.9]	0.985 [0.977–0.993]	98.2% [97.9–98.5]	85.3% [82.3–88.3]	99.2% [99.1–99.4]
End-to-end: vein segmentation and classification	84.1% [82.3–85.9]	0.978 [0.971–0.986]	97.7% [97.3–98.1]	82.8% [78.0–87.6]	99.0% [98.7–99.3]

AUC = area under the receiver operating characteristic curve; CI = confidence interval.

To isolate the performance of the classification model from segmentation errors, Stage 2 was evaluated exclusively within ground-truth vessel regions. As shown in Table 1, the classifier achieved Dice coefficients of 96.3% for arteries and 96.1% for veins, with AUC values of 0.991 and 0.992, respectively. The high sensitivity for arteries and veins, 0.950 and 0.980, respectively, indicates that the model reliably distinguishes vessel identity even in the absence of first- and second-order vascular landmarks.

The combined pipeline (Fig 2), in which the binary segmentation mask from Stage 1 gates the classification output from Stage 2, represents the clinically relevant end-to-end performance. The system achieved Dice coefficients of 87.3% for arteries and 84.1% for veins, with AUC values of 0.985 and 0.978, respectively. Specificity exceeded 0.989 for both vessel classes, reflecting precise spatial localization with minimal cross-class contamination.

**Figure 2:**
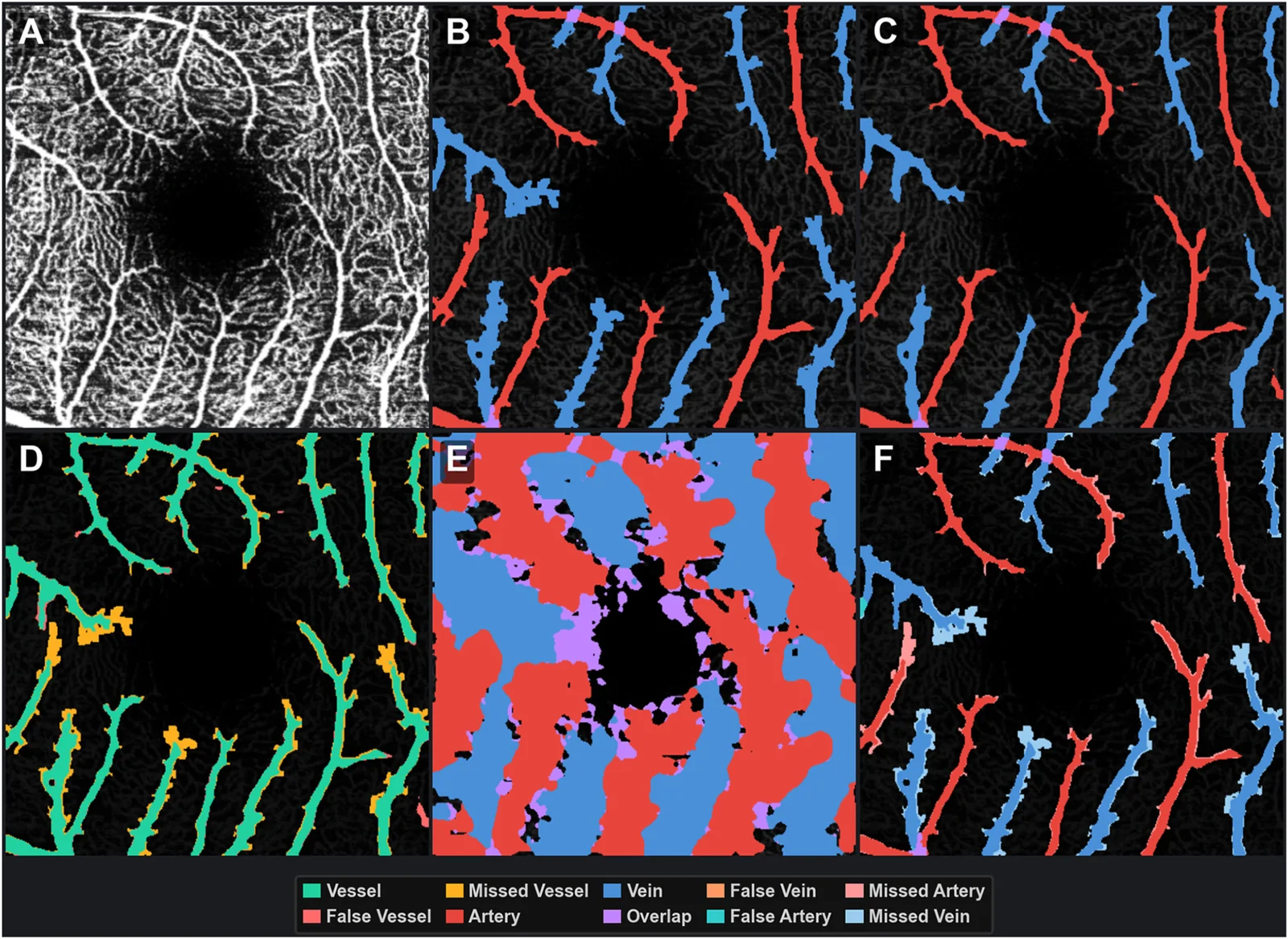
Combined end-to-end system performance. Combined end-to-end AV segmentation and classification results for the representative holdout test eye. **(A)** Raw en face OCT angiography image. **(B)** Ground truth AV labels. **(C)** Final predicted AV map produced by gating the stage 2 classification output with the stage 1 binary segmentation mask. **(D)** Stage 1 segmentation error map showing correct, false, and missed vessel detections. **(E)** Stage 2 predicted classification map showing the raw artery, vein, and overlap assignments prior to segmentation gating. **(F)** End-to-end combined error map illustrating the end-to-end system performance, where correctly identified arteries and veins retain ground truth colors and errors are distinguished by type. AV = artery-vein.

### RVO

The model was applied to the RVO pilot cohort without retraining. To assess generalizability to pathological vasculature, expert AV ground truth was generated for all 9 RVO scans. Across these scans, Stage 1 vessel segmentation achieved a mean Dice coefficient of 82.5% ± 3.1% [95% CI, 80.2%–84.8%]. End-to-end AV classification yielded mean Dice coefficients of 75.6% ± 4.4% [95% CI, 72.3%–79.0%] for arteries and 76.8% ± 5.2% [95% CI, 72.9%–80.8%] for veins, with specificity exceeding 97.9% for both vessel classes (artery: 98.2% ± 0.4%; vein: 97.9% ± 1.1%). These values represent an expected reduction from healthy-eye performance (end-to-end Dice: 87.3% arteries, 84.1% veins), consistent with the increased complexity of pathological vasculature. Importantly, high specificity was maintained, indicating that the model reliably avoids misclassifying vessel identity even when segmentation sensitivity is reduced on diseased eyes.

### Vascular Biomarker Extraction

The model's predicted AV maps yielded 2 statistically independent signals consistent with established RVO pathophysiology (Table 2) (Fig 3). Global branches include all arteries and veins that were at least 62 μm, or approximately 5% of the length and width of the scan. This was not a preferred measure, as the capillary bed can have outsized effects and there is no agreement on criteria for determining if a capillary branch is more arteriolar or venular. Hence, primary end points were restricted to major vessels, which are defined as at least 310 μm, or approximately 10% of the length and width of the scan for which agreement on criteria determining if a vessel is an artery or vein exists.^32^ The major vein/artery sinuosity ratio was significantly elevated in RVO compared with controls (1.041 vs. 1.010; difference, +3.1%; *P* = 0.007; AUC, 0.837 [95% CI, 0.60–1.00]). In healthy eyes, arteries and veins exhibit nearly balanced sinuosity (ratio ≈ 1.0). In RVO, veins became selectively more tortuous, a well-documented clinical hallmark of venous outflow obstruction, while major arteries remained geometrically unchanged (major artery sinuosity index: 1.110 vs. 1.111; *P* = 0.37). Independently, the 10th-percentile artery radius was significantly narrower in RVO (13.2 μm vs. 15.4 μm; difference, –14.8%; *P* = 0.002; AUC, 0.885 [95% CI, 0.72–1.00]). Mean artery and vein radii were not significantly different between groups (*P* > 0.10 for both), indicating that the attenuation signal is concentrated in the smallest arterioles rather than the major trunks, and most likely reflects coexisting hypertensive and arteriosclerotic vascular disease rather than a direct hemodynamic consequence of the venous occlusion, given that all RVO patients in this cohort were hypertensive. Focal arteriolar narrowing is an established correlate of both hypertension and RVO in population studies.^33^^,^^34^

**Table 2:** Vascular Biomarker Extraction: RVO vs. Control

Metric	RVO (*n* = 9)	Control (n = 15)	Δ	*P*	AUC [95% CI]
Primary end points					
Major vein/artery sinuosity	1.041	1.010	+3.1%	0.007	0.837 [0.60–1.00]
10th-percentile artery radius (μm)	13.2	15.4	–14.8%	0.002	0.885 [0.72–1.00]
Secondary end points					
Global vein/artery sinuosity	1.030	1.006	+2.3%	0.020	0.793 [0.59–0.96]
Major vein sinuosity index	1.156	1.122	+3.0%	0.074	—
Major artery sinuosity index	1.110	1.111	–0.1%	0.371	—
Global vein sinuosity index	1.139	1.113	+2.4%	0.136	—
Global artery sinuosity index	1.107	1.106	0.0%	0.325	—

AUC = area under the receiver operating characteristic curve; RVO = retinal vein occlusion; CI = confidence interval.

All images 3 × 3 mm^2^. Two-sided Mann–Whitney *U*. Area under the receiver operating characteristic curve 95% CI: bootstrap (2000 resamples). Branch thresholds defined in physical units, converted per image from known field of view: major branches ≥310 μm; global branches ≥ 62 μm. RVO (*n* = 9) and healthy control images (*n* = 15) at approximately 960 × 960 pixels were included. Model applied without retraining.

**Figure 3:**
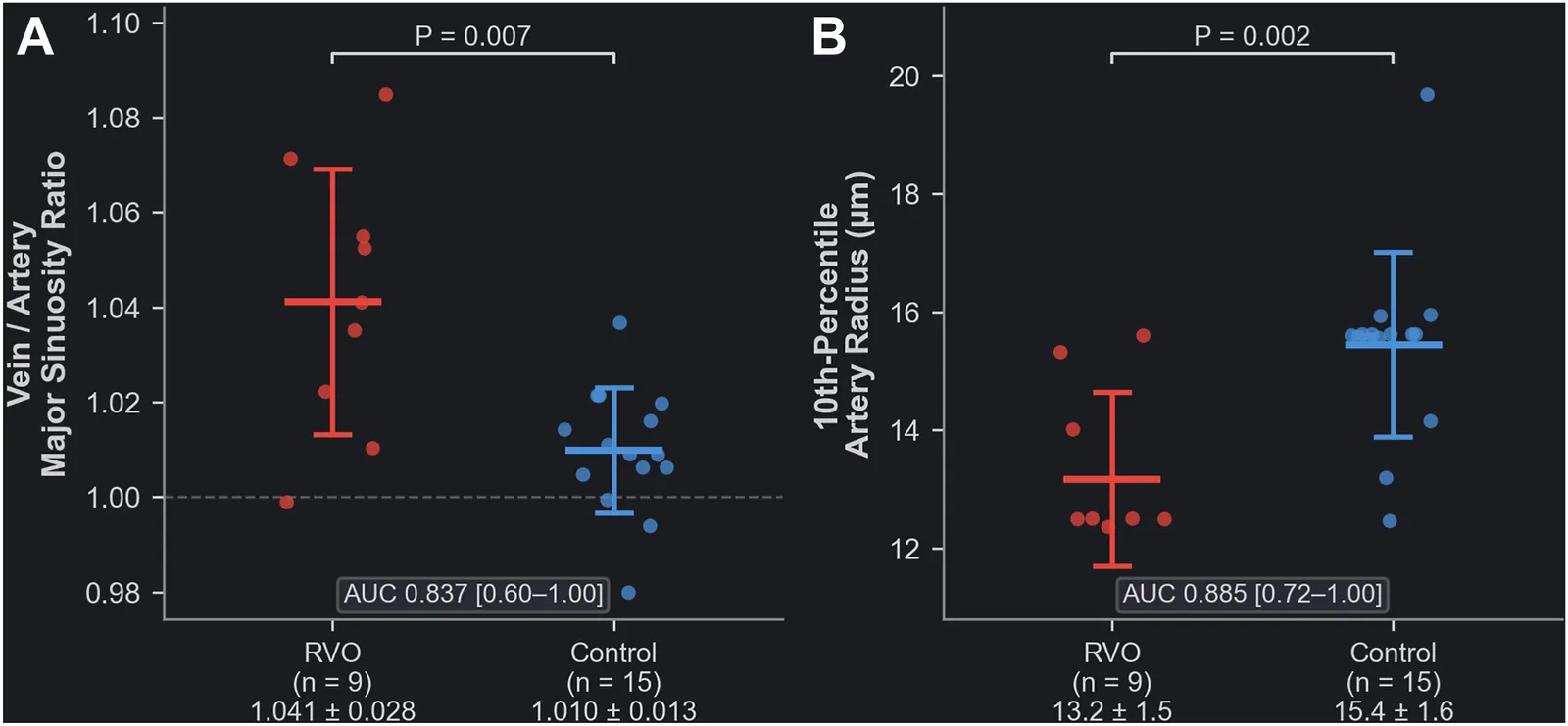
Vascular biomarker distributions in RVO vs. healthy controls. Each panel shows individual image-level values (jitter plot) with mean ± standard deviation. **(A)** Major vein/artery sinuosity ratio. In healthy eyes, arterial and venous tortuosity are nearly balanced (ratio ≈ 1.0; dashed reference line). In RVO, veins become selectively more tortuous than arteries within the same eye (*P* = 0.007; AUC = 0.837 [95% CI, 0.60–1.00]). **(B)** Tenth-percentile artery radius. The thinnest arteriolar segments are significantly narrower in RVO (13.2 μm) than in controls (15.4 μm), a 14.8% attenuation more likely reflecting coexisting hypertensive vascular disease than the occlusion itself (*P* = 0.002; AUC = 0.885 [95% CI, 0.72–1.00]). AUC = area under the receiver operating characteristic curve; RVO = retinal vein occlusion; CI = confidence interval.

## Discussion

We presented a 2-stage Swin-Unet framework that successfully differentiates arteries from veins in small-field macular OCTA scans using a training set of 12 eyes. By decoupling the structural segmentation of vessels from their physiological classification, our system achieved end-to-end Dice coefficients of 87.3% for arteries and 84.1% for veins. These results demonstrate that incorporating a geometric prior, specifically a Frangi vesselness filter, allows a transformer architecture to compensate for the missing global spatial cues that typically make analyzing foveal scans difficult.

The primary challenge addressed in this study is achieving data-efficient AV differentiation that remains robust in pathological states. This is particularly difficult in foveal-centered 3 × 3-mm^2^ scans due to their inherent “landmark deficiency.” Most existing OCTA segmentation models such as OCTA-ReVA + AV or AVA-Net^28^^,^^35^ are optimized for wider fields of view where first- and second-order vessels are clearly visible. These macrostructures provide distinct caliber, color, and orientation cues that act as global anchors for classification. In contrast, small-field macular scans are dominated by fourth- and fifth-order branches embedded in a dense, noisy capillary mesh. In this environment, the morphological hierarchy used by traditional CNNs largely disappears. As outlined in the Introduction, this loss of discriminative cues favors a transformer-based approach where true direct global context enables the ability to integrate spatial context across the entire image. We found that the Swin transformer backbone is uniquely suited to this task. Unlike standard CNNs that rely on indirect skip connections and local receptive fields, the shifted-window attention mechanism of the transformer directly models long-range dependencies across the entire macula. This allows the network to maintain vessel identity by tracing continuous paths across disjointed segments, effectively reconstructing the missing global context through learned spatial relationships. By leveraging a transformer architecture, we achieve comparable performance on major vessel classification and segmentation to MF-AV-Net^19^ and OCTA-ReVA + AV^28^ with an order of magnitude less training data (150 dual modality OCTA scans vs. 12 OCTA scans).

Beyond technical classification and segmentation, reliable automated separation of these vascular systems enables precise quantification of remodeling specific to a given disease. As proof of concept, we applied our trained model to a pilot cohort of 9 patients with RVO and 15 controls without retraining it on pathological data. The model successfully recovered 2 independent biological signals. First, the major vein-to-artery sinuosity ratio was significantly elevated in RVO (1.041 vs. 1.010), reflecting selective venous tortuosity. Because isolated artery and vein sinuosity alone were not significantly altered, the discriminating signal resided entirely in the arteriovenous imbalance. We captured this by using the arteries of each patient as an internal reference to standardize the measurement. Second, the lowest 10% of artery radii were narrower in RVO (13.2 μm vs. 15.4 μm), while the mean artery radius remained unchanged. This precisely isolates the selective attenuation of the smallest arterioles, most likely reflecting the systemic hypertensive and arteriosclerotic disease strongly associated with RVO rather than a hemodynamic consequence of the occlusion itself^33^^,^^34^; this caliber end point is therefore more susceptible to systemic confounding than the within-eye vein/artery sinuosity ratio, which standardizes against each patient’s own arteries. Unlike vessel density, which can be confounded by signal artifacts, the major vein-to-artery sinuosity ratio provides a geometrical invariant directly linked to hemodynamic stress. This adds a layer of clinical authority to the metric and demonstrates the power of vessel-specific biomarkers over global averages. These findings suggest a potential scalable pathway for deploying site-specific models in clinical workflows, though this requires prospective validation.

From a practical perspective, a major outcome of this study is the demonstration of data-efficient learning in a clinical setting. Deep-learning tools for OCTA are frequently bottlenecked by the need for massive expert-annotated datasets. For comparison, the AVA-Net^35^ model developed by Abtahi et al, was trained on over 100 images to achieve its strong performance. Our pipeline achieved competitive end-to-end fidelity under harsher capillary conditions in a smaller field-of-view using a training set of 12 eyes—an order of magnitude fewer annotated examples. This implies a scalable pathway for deploying site-specific models in clinics without the need for prohibitive data curation.

This study has several limitations. First, our development cohort was small and restricted to healthy eyes. Validation on the 9-scan RVO cohort demonstrated that the model generalizes to pathological vasculature with an expected reduction in performance (end-to-end Dice: 75.6% arteries, 76.8% veins vs. 87.3% and 84.1% on healthy eyes), while maintaining high specificity (>97.9%). This performance gap likely reflects the increased vascular complexity and morphological distortion inherent to diseased vasculature. Critically, however, this modest data requirement is itself a strength of the data-efficient design rather than only a constraint: should accuracy decline for a given disease, the data-efficient paradigm allows the model to be retrained on a limited number of pathologic images rather than the hundreds typically required by conventional approaches. Second, our ground truth relied on sparse manual annotations. This was intentional, as at levels beyond the fourth- and fifth-order arterioles and venules, there is no established methodology for determining if a capillary is more arteriolar or venular. Finally, the RVO biomarker analysis is an exploratory pilot and does not constitute a formal diagnostic accuracy study; these findings should be considered hypothesis-generating. Although the model demonstrated robust performance on our internal test set, it remains trained on data from a single institution (Washington University) and platform (Heidelberg Spectralis). Prospective validation on multicenter, multiplatform datasets is required to confirm true generalizability beyond the current cohort. While the 2-stage architecture improves accuracy, it inherently increases computational complexity compared to single-stage baselines. Future work will focus on validating this framework on distinct retinal pathologies and exploring semisupervised learning to leverage the vast amounts of unlabeled OCTA data available in clinical repositories.

Furthermore, the RVO pilot cohort is limited by uncontrolled confounders; patients with RVO have concurrent hypertension and are likely older than the healthy control group. As these factors are known to influence vascular caliber and tortuosity, the biomarker findings should be interpreted as hypothesis-generating only.

This study presents a data-efficient, 2-stage transformer framework for automated AV differentiation in landmark-deficient macular OCTA. By decoupling vessel segmentation from classification and incorporating a Frangi vesselness prior, the model achieved end-to-end Dice coefficients of 87.3% (arteries) and 84.1% (veins) using a training set of 12 eyes. Applied to a pilot RVO cohort, the model successfully recovered biologically independent vascular biomarkers, detecting the distinct venous tortuosity caused by venous outflow obstruction and raised venous back pressure without requiring retraining. This highly scalable framework eases the bottleneck of exhaustive expert annotation, offering a robust tool for vessel-specific quantification in clinical research. Future efforts will validate the model in pathological states, such as diabetic retinopathy, and explore semisupervised learning to leverage unannotated OCTA repositories.

## Declaration of Generative AI and AI-Assisted Technologies in the Writing Process

During the preparation of this work, the authors utilized Gemini and a self-hosted instance of Qwen3-Coder-32B to help with code organization, refactoring, and readability, with all changes manually verified. Following the use of these tools, the authors reviewed and edited the content as needed and take full responsibility for the integrity and accuracy of the publication.
