# Supplementary material for: Data-Efficient Transformer Model for Retinal Artery-Vein Segmentation in Landmark-Deficient OCT Angiography
Source: Ophthalmol Sci. 2026 Sep 23;6(12):101416. doi: 10.1016/j.xops.2026.101416 (PMC13734878; doi:10.1016/j.xops.2026.101416)
Supplement: Supplementary file 1 [file mmc1.docx]

Supplementary Table 3. Frangi Vesselness Filter Ablation

| **Method** | **Mean Dice** | **Mean Sensitivity** | **Mean Specificity** |
| --- | --- | --- | --- |
| DL_Stage1_SwinUnet | 0.8852 | 0.8663 | 0.9853 |
| Frangi_wide_lenient+otsu | 0.3775 | 0.6383 | 0.7023 |
| Frangi_lenient+otsu | 0.3775 | 0.6384 | 0.7022 |
| Frangi_strict+percentile_90 | 0.365 | 0.7309 | 0.612 |
| Frangi_default+percentile_90 | 0.3614 | 0.7535 | 0.5882 |
| Frangi_wide+percentile_90 | 0.3614 | 0.7604 | 0.5826 |
| Frangi_strict+percentile_95 | 0.3612 | 0.7595 | 0.5825 |
| Frangi_wide+percentile_95 | 0.3585 | 0.7884 | 0.5548 |
| Frangi_default+percentile_95 | 0.3575 | 0.7712 | 0.5663 |
| Frangi_lenient+adaptive | 0.3571 | 0.6398 | 0.669 |
| Frangi_wide_lenient+adaptive | 0.357 | 0.6392 | 0.6692 |
| Frangi_wide_lenient+percentile_90 | 0.3545 | 0.8423 | 0.5037 |
| Frangi_wide_lenient+percentile_95 | 0.3528 | 0.8696 | 0.4776 |
| Frangi_lenient+percentile_90 | 0.3526 | 0.7897 | 0.5416 |
| Frangi_lenient+percentile_95 | 0.3469 | 0.8082 | 0.5138 |
| Frangi_CLAHE_lenient+otsu | 0.3465 | 0.6535 | 0.6391 |
| Frangi_CLAHE+percentile_90 | 0.3464 | 0.7812 | 0.5351 |
| Frangi_CLAHE+percentile_95 | 0.346 | 0.8068 | 0.5132 |
| Frangi_default+adaptive | 0.3456 | 0.5095 | 0.7555 |
| Frangi_wide+adaptive | 0.3456 | 0.5094 | 0.7555 |
| Frangi_CLAHE_lenient+percentile_90 | 0.3456 | 0.8175 | 0.5036 |
| Frangi_CLAHE_lenient+percentile_95 | 0.3418 | 0.8421 | 0.4743 |
| Frangi_strict+adaptive | 0.3395 | 0.4745 | 0.7757 |
| Frangi_wide+otsu | 0.3381 | 0.4309 | 0.8121 |
| Frangi_default+otsu | 0.3381 | 0.4309 | 0.8121 |
| Frangi_CLAHE_lenient+adaptive | 0.3362 | 0.6509 | 0.621 |
| Frangi_strict+otsu | 0.313 | 0.366 | 0.838 |
| Frangi_CLAHE+adaptive | 0.3101 | 0.5004 | 0.706 |
| Frangi_CLAHE+otsu | 0.2875 | 0.4086 | 0.7617 |

*Values represent the mean across all validation scans. DL_Stage1_SwinUnet significantly outperforms all thresholding-based Frangi configurations.*
